# Supplementary material for: A coma pattern-based autofocusing method resolves bacterial cold shock response at single-cell level
Source: eLife. 2026 Jul 6;15:RP110268. doi: 10.7554/eLife.110268 (PMC13336770; doi:10.7554/eLife.110268)
Supplement: Peer review history

# Editor report: eLife Assessment

**DOI:** 10.7554/eLife.110268.3.sa0

**Authors:** Felix Campelo

**Affiliations:** Universitat Pompeu Fabra Spain

**Keywords:** Convincing, Important

This **important** study introduces LUNA, a new autofocusing method that achieves nanoscale precision and robustly corrects focus drift during time-lapse microscopy, improving imaging under temperature shifts. The authors exploit this technical advance to investigate the bacterial cold shock response, providing **convincing** evidence that individual cells continue to grow and divide in a highly coordinated process that cannot be observed in population-level measurements. This work offers a technical and conceptual framework for reconciling discrepancies between bulk and single-cell growth measurements, with broad relevance for cell biology and microbiology.

# Referee report: Reviewer #1 (Public review)

**DOI:** 10.7554/eLife.110268.3.sa1

Summary:

The authors present a new autofocusing method, LUNA (Locking Under Nanoscale Accuracy), designed to overcome severe focus drift, a major challenge in long-term time-lapse microscopy. Using this method, they address a fundamental question in bacterial cold shock response: whether cells halt growth and division following an abrupt temperature downshift. Through single-cell analysis, the authors uncover a multi-phase adaptation process with distinct growth deceleration dynamics, and show that bacterial cells adapt to cold shock in a largely uniform manner across the population. Overall, this work provides new insights into the bacterial cold shock response at the single-cell level, extending beyond what can be inferred from population-level measurements.

Strengths:

(1) The LUNA method shows improved performance compared to existing autofocusing systems, achieving nanoscale precision over a large focusing range. Its focusing speed is sufficient for the experiments presented, with potential for further improvement through faster motors and optimized control algorithms, suggesting broad applicability. Theoretical simulations and experimental validation together provide strong support for the method's robustness.

(2) Using LUNA, the authors address a long-standing question in bacterial physiology: whether cells arrest growth and division during the acclimation phase following cold shock. Single-cell analyses across the full course of cold adaptation reveal features that are obscured in bulk-culture studies. Cells continue to grow and divide at reduced rates while maintaining cell size regulation, and exhibit a three-phase adaptation program with distinct growth dynamics. This response appears uniform across the population, with no evidence for bet-hedging. Overall, the experiments are well designed, and the analyses are solid and support the authors' conclusions.

(3) The authors further propose a model describing how population-level optical density (OD) depends on cell dry mass density, volume, and concentration. Following cold shock, cells grow more slowly and exhibit smaller sizes, explaining the apparently unchanged OD. This model provides a valuable conceptual framework for interpreting OD-based growth measurements, a widely used method in microbiology, and will be of broad interest to the field.

Weaknesses:

No major weaknesses identified.

Comments on revisions:

The authors have thoroughly addressed all of my questions. I thank them for their clear clarifications and thoughtful revisions, and I greatly appreciate their efforts in improving the manuscript.

# Referee report: Reviewer #2 (Public review)

**DOI:** 10.7554/eLife.110268.3.sa2

Summary:

This study presents LUNA, an autofocus method that compensates for focus drift during rapid temperature changes. Using this approach, the authors show that *E. coli* cells continue to grow and divide during cold shock, revealing a coordinated, multi-phase adaptation process that could not be deduced from traditional population measurements. They propose a scattering-theory-based model that reconciles the paradox between growth differences of the bacteria at the single-cell level vs population level.

Strengths:

(1) The LUNA approach is pretty creative, turning coma aberration from what is normally a nuisance into an exploit. LUNA enabled long-term single-cell imaging during rapid temperature downshifts.

(2) The authors show that the long-assumed growth arrest during cold shock from population-level measurements is misleading. At the single-cell level, bacteria do not stop growing or dividing but undergo a continuous, three-phase adaptation process. Importantly, this behavior is highly synchronized across the population and not based on bet-hedging.

(3) Finally, the authors propose a model to resolve a long-standing paradox between single-cell vs population behavior: if cells keep growing, why does optical density (OD) of the culture stop increasing? Using light-scattering theory, they show that OD depends not only on cell number but also on cell volume, which decreases after cold shock. As a result, OD can remain flat, or even decrease, despite continued biomass accumulation. This demonstrates that OD is not a reliable proxy for growth under non-steady conditions.

Weaknesses:

(1) While the authors theoretically explain the advantages of LUNA over existing autofocus methods, it is unclear whether practical head-to-head comparisons have been performed, apart from the comparison to Nikon PFS shown in Video S1. As written, the manuscript gives the impression that only LUNA can solve this problem, but such a claim would require more systematic and rigorous benchmarking against alternative approaches.

(2) No mutants/inhibitors used to test and challenge the proposed model.

(3) Cells display a high degree of synchronization, but they are grown in confined microfluidic channels under highly uniform conditions. It is unclear to what extent this synchrony reflects intrinsic biology versus effects imposed by the microfluidic environment.

(4) To further test and generalize the model, it would be informative to also examine bacterial responses at intermediate temperatures rather than focusing primarily on a single cold-shock condition.

Comments on revisions:

The authors have addressed my comments in their response, but have chosen not to incorporate most of them into the manuscript. Readers may refer to the peer review section for further details.

# Author comment: Author response

**DOI:** 10.7554/eLife.110268.3.sa3

**Authors:** Shuqiang Huang, Sihong Li, Zhixin Ma, Yue Yu, Jinjuan Wang, Yaxin Shen, Xiaodong Cui, Xiongfei Fu

**Affiliations:** Shenzhen Institutes of Advanced Technology Shenzhen China; Shenzhen Institutes of Advanced Technology Shenzhen China; State Key Laboratory of Quantitative Synthetic Biology, Shenzhen Institute of Synthetic Biology, Shenzhen Institutes of Advanced Technology, Chinese Academy of Sciences Shenzhen China; Shenzhen Institutes of Advanced Technology Shenzhen China; Shenzhen Institutes of Advanced Technology Shenzhen China; Shenzhen Institutes of Advanced Technology Shenzhen China; University of Hong Kong Hong Kong Hong Kong; Shenzhen Institutes of Advanced Technology, Chinese Academy of Sciences Shenzhen China

The following is the authors’ response to the current reviews.

**Public Reviews:**

**Reviewer #1 (Public review):**

Summary:

The authors present a new autofocusing method, LUNA (Locking Under Nanoscale Accuracy), designed to overcome severe focus drift, a major challenge in long-term time-lapse microscopy. Using this method, they address a fundamental question in bacterial cold shock response: whether cells halt growth and division following an abrupt temperature downshift. Through single-cell analysis, the authors uncover a multi-phase adaptation process with distinct growth deceleration dynamics, and show that bacterial cells adapt to cold shock in a largely uniform manner across the population. Overall, this work provides new insights into the bacterial cold shock response at the single-cell level, extending beyond what can be inferred from population-level measurements.

Strengths:

(1) The LUNA method shows improved performance compared to existing autofocusing systems, achieving nanoscale precision over a large focusing range. Its focusing speed is sufficient for the experiments presented, with potential for further improvement through faster motors and optimized control algorithms, suggesting broad applicability. Theoretical simulations and experimental validation together provide strong support for the method's robustness.

(2) Using LUNA, the authors address a long-standing question in bacterial physiology: whether cells arrest growth and division during the acclimation phase following cold shock. Single-cell analyses across the full course of cold adaptation reveal features that are obscured in bulk-culture studies. Cells continue to grow and divide at reduced rates while maintaining cell size regulation, and exhibit a three-phase adaptation program with distinct growth dynamics. This response appears uniform across the population, with no evidence for bet-hedging. Overall, the experiments are well designed, and the analyses are solid and support the authors' conclusions.

(3) The authors further propose a model describing how population-level optical density (OD) depends on cell dry mass density, volume, and concentration. Following cold shock, cells grow more slowly and exhibit smaller sizes, explaining the apparently unchanged OD. This model provides a valuable conceptual framework for interpreting OD-based growth measurements, a widely used method in microbiology, and will be of broad interest to the field.

Weaknesses:

No major weaknesses identified.

Comments on revisions:

The authors have thoroughly addressed all of my questions. I thank them for their clear clarifications and thoughtful revisions, and I greatly appreciate their efforts in improving the manuscript.

We sincerely thank the reviewer’s for the encouraging comments and positive assessment. We greatly appreciate the reviewer’s constructive feedback during the review process, which helped us improve the manuscript.

**Reviewer #2 (Public review):**

Summary:

This study presents LUNA, an autofocus method that compensates for focus drift during rapid temperature changes. Using this approach, the authors show that *E. coli* cells continue to grow and divide during cold shock, revealing a coordinated, multi-phase adaptation process that could not be deduced from traditional population measurements. They propose a scattering-theory-based model that reconciles the paradox between growth differences of the bacteria at the single-cell level vs population level.

Strengths:

(1) The LUNA approach is pretty creative, turning coma aberration from what is normally a nuisance into an exploit. LUNA enabled long-term single-cell imaging during rapid temperature downshifts.

(2) The authors show that the long-assumed growth arrest during cold shock from population-level measurements is misleading. At the single-cell level, bacteria do not stop growing or dividing but undergo a continuous, three-phase adaptation process. Importantly, this behavior is highly synchronized across the population and not based on bet-hedging.

(3) Finally, the authors propose a model to resolve a long-standing paradox between single-cell vs population behavior: if cells keep growing, why does optical density (OD) of the culture stop increasing? Using light-scattering theory, they show that OD depends not only on cell number but also on cell volume, which decreases after cold shock. As a result, OD can remain flat, or even decrease, despite continued biomass accumulation. This demonstrates that OD is not a reliable proxy for growth under non-steady conditions.

Weaknesses:

(1) While the authors theoretically explain the advantages of LUNA over existing autofocus methods, it is unclear whether practical head-to-head comparisons have been performed, apart from the comparison to Nikon PFS shown in Video S1. As written, the manuscript gives the impression that only LUNA can solve this problem, but such a claim would require more systematic and rigorous benchmarking against alternative approaches.

(2) No mutants/inhibitors used to test and challenge the proposed model.

(3) Cells display a high degree of synchronization, but they are grown in confined microfluidic channels under highly uniform conditions. It is unclear to what extent this synchrony reflects intrinsic biology versus effects imposed by the microfluidic environment.

(4) To further test and generalize the model, it would be informative to also examine bacterial responses at intermediate temperatures rather than focusing primarily on a single cold-shock condition.

Comments on revisions:

The authors have addressed my comments in their response, but have chosen not to incorporate most of them into the manuscript. Readers may refer to the peer review section for further details.

We thank the reviewer for this additional comments and for the careful suggestions, and we appreciate that the raised points are valuable for a broader discussion of the topic. In the revised manuscript, we have incorporated the comments most directly relevant to the scope and central conclusions of the study, and have clarified these points in the text where appropriate. Specifically, we have clarified several key issues, including the interpretation of the OD lag as a “combined effect,” the performance and application scope of LUNA, the alignment of cell-cycle progression after cold shock, and relevant methodological details.

For the remaining contextual issues, we have kept the detailed discussion in the response to reviewers rather than expanding the manuscript extensively, so as to preserve the focus and readability of the main text. We hope that the revisions now better acknowledge the reviewer’s concerns while maintaining a concise presentation of the central findings.

The following is the authors’ response to the original reviews.

**Public Reviews:**

**Reviewer #1 (Public review):**

Summary:

The authors developed a new autofocusing method, LUNA (Locking Under Nanoscale Accuracy), to address severe focus drift-a major challenge in time-lapse microscopy. Using this method, they tackle a fundamental question in bacterial cold shock whether cells halt growth and division following an abrupt temperature downshift. Overall, the experimental design, modeling, and data analysis are solid and well executed. However, several points require clarification or further support to fully substantiate the authors' conclusions.

Strengths:

(1) The LUNA method outperforms existing autofocusing systems with nanoscale precision over a large focusing range. The focusing time is reasonable for the presented experiments, and the authors note potential improvements by using faster motors and optimized control algorithms, suggesting broad applicability. The theoretical simulations and experimental validation provide solid support for the robustness of the method.

(2) Using LUNA, the authors address a long-standing question in bacterial physiology: whether cells arrest growth and division after an abrupt cold shock. Single-cell analyses monitoring the entire course of cold adaptation and steady-state growth reveal features that are obscured in bulk-culture studies: cells continue to grow at reduced rates with smaller cell sizes, resulting in an apparently unchanged population-level OD. The experiments are well designed and analyses are generally solid and largely support the authors' conclusions.

(3) The authors also propose a model describing how population-level OD measurements depend on cell dry mass density, volume, and concentration. This provides a valuable conceptual contribution to the interpretation of OD-based growth measurements, which remain a gold-standard method in microbiology.

We thank the reviewer for acknowledging the strengths of our study.

Weaknesses:

(1) It is unclear whether the author's model explaining the population-level OD during acclimation is broadly applicable. Most analyses focus on a shift from 37°C to 14°C, where the model agrees well with experimental data. However, in the 37°C to 12°C experiment, OD600 decreases after cold shock (Fig. 5e), and the computed OD does not match the experimental measurements (Fig. S16a). Although the authors attribute this discrepancy to a "complicated interplay," no further explanation is provided, which limits confidence in the model's general applicability.

Thank you for this careful evaluation regarding the model generality. In the experiment with a temperature shift from 37°C to 12°C, the measured OD600 values were 0.243 at 0 hours and 0.242 at 5 hours. In comparison, our model-computed OD600 values were 0.243 at 0 hours and 0.271 at 5 hours. The absolute difference between the measured and computed values at 5 hours is therefore 0.028.

Given the typical experimental variability in OD600 measurements and the limited linear range of the OD-to-biomass approximation (generally considered reliable below ~0.5), this deviation is quantitatively modest. We appreciate your valuable feedback and are happy to provide further clarification if needed.

(2) The manuscript proposes that cell-cycle progression becomes synchronized across the population after cold shock, but the supporting evidence is not fully convincing. If synchronization refers primarily to the uniform reduction in growth rate following cold shock, this could plausibly arise from global translation inhibition affecting all cells. However, the additional claim that "cells encountering a relatively late CSR will accelerate division to maintain synchronization" is not strongly supported by the presented data.

We appreciate your critical reading, which has helped us identify ambiguities in our terminology and strengthen the clarity of our work. Regarding the term “synchronization”, we would like to clarify that it refers to two different scenarios: (i) the synchrony in the timing of growth rate changes after cold shock. The cells initiate the slowdown in growth almost simultaneously, suggesting a highly coordinated, non-stochastic population-level response to cold shock; (ii) the synchrony in division cycle progression.

In the sentence you referenced “cells encountering a relatively late CSR will accelerate divisions to maintain synchronization”, we intended to describe that cells maintain consistent progression of the division cycle after cold shock, meaning that after the same number of elapsed cycles, different cells are at a similar stage in their division timing (Figure 4f, 4g, Figure S14). The term “accelerate” refers to our observation that cells which complete a given cycle later than others tend to have shorter subsequent inter-division intervals, thereby “catching up” to maintain alignment in cycle number across the population. We acknowledge that using “synchronization” in this scenario may be ambiguous, and we will replace it with more precise phrasing “progression of division cycle” to accurately convey this finding.

(3) Several technical terms used in the method development section are not clearly defined and may be unfamiliar to a broad readership, which makes it difficult to fully understand the methodology and evaluate its performance. Examples include depth of focus, focusing precision, focusing time, focusing frequency, and drift threshold value. In addition, the reported average focusing time per location (~0.6 s) lacks sufficient context, limiting the reader's ability to assess its significance relative to existing autofocusing methods.

Thank you for your valuable comments and suggestions. In response, we have added more detailed descriptions in the Methods section of the revised version.

The reviewer noted that the reported average focusing time (~0.6 s) lacks sufficient context, which may limit readers’ ability to assess its significance relative to existing autofocusing methods. We would like to clarify that the core innovation of this work lies in the proposed theoretical framework for autofocusing, which offers advantages over existing methods in terms of focusing precision and range. While focusing time is a practically relevant performance metric, it is primarily presented here as an implementation-dependent parameter rather than a central theoretical contribution of this study. In our experimental setup, an average focusing time of 0.6 s proved sufficient for routine timelapse imaging in microscopy, thereby demonstrating the practical usability of LUNA.

**Reviewer #2 (Public review):**

Summary:

This study presents LUNA, an autofocus method that compensates for focus drift during rapid temperature changes. Using this approach, the authors show that *E. coli* cells continue to grow and divide during cold shock, revealing a coordinated, multi-phase adaptation process that could not be deduced from traditional population measurements. They propose a scattering-theory-based model that reconciles the paradox between growth differences of the bacteria at the single-cell level vs population level.

Strengths:

(1) The LUNA approach is pretty creative, turning coma aberration from what is normally a nuisance into an exploit. LUNA enabled long-term single-cell imaging during rapid temperature downshifts.

(2) The authors show that the long-assumed growth arrest during cold shock from population-level measurements is misleading. At the single-cell level, bacteria do not stop growing or dividing but undergo a continuous, three-phase adaptation process. Importantly, this behavior is highly synchronized across the population and not based on bet-hedging.

(3) Finally, the authors propose a model to resolve a long-standing paradox between single-cell vs population behavior: if cells keep growing, why does optical density (OD) of the culture stop increasing? Using light-scattering theory, they show that OD depends not only on cell number but also on cell volume, which decreases after cold shock. As a result, OD can remain flat, or even decrease, despite continued biomass accumulation. This demonstrates that OD is not a reliable proxy for growth under non-steady conditions.

We thank the reviewer for acknowledging the strengths of our study.

Weaknesses:

(1) While the authors theoretically explain the advantages of LUNA over existing autofocus methods, it is unclear whether practical head-to-head comparisons have been performed, apart from the comparison to Nikon PFS shown in Video S1. As written, the manuscript gives the impression that only LUNA can solve this problem, but such a claim would require more systematic and rigorous benchmarking against alternative approaches.

Thank you for your insightful comment regarding the comparison of LUNA with other autofocus methods.

In our study, we primarily compared LUNA with the Nikon PFS system (as shown in Video S1) because Nikon PFS is one of the most widely used commercial autofocus systems in single-cell time-lapse imaging, and its manufacturer provides well-defined performance parameters (e.g., focusing precision within 1/3 depth-of-focus, response time <0.7 s), which facilitates a quantitative comparison. For other commercial systems, such as Olympus ZDC, Zeiss Definite Focus, Leica AFC, and ASI CRISP, the publicly available specifications are often less clearly defined, or are measured under inconsistent conditions, making a direct head-to-head comparison challenging and potentially misleading. Additionally, in our preliminary experiments, we also tested an Olympus microscope and observed severe focus drift during slow cooling processes. From a physical perspective, LUNA is specifically designed to meet the demanding requirements of single-cell experiments, including a wide focusing range and high precision, while existing commercial systems may not physically achieve the combination of range and accuracy needed for such extreme conditions.

(2) No mutants/inhibitors used to test and challenge the proposed model.

We agree that such approaches would provide valuable mechanistic insights and further strengthen the validation of the model presented in this study. In the current work, our primary goal was to introduce LUNA autofocusing method and demonstrate its capability to resolve bacterial cold shock response at the single-cell level with unprecedented precision. As such, we focused on characterizing the wild-type physiological dynamics under cold shock, which already revealed several previously unreported phenomena. We acknowledge that the use of genetic mutants or chemical inhibitors targeting specific cold shock proteins or regulatory pathways would be a logical and powerful next step to dissect the underlying molecular mechanisms and test the causality of the observed growth dynamics. We plan to address this in future work by incorporating such perturbations to further test and refine the model.

(3) Cells display a high degree of synchronization, but they are grown in confined microfluidic channels under highly uniform conditions. It is unclear to what extent this synchrony reflects intrinsic biology versus effects imposed by the microfluidic environment.

The reviewer raises a pertinent question regarding whether the observed high degree of cell synchronization represents an intrinsic biological phenomenon or an artifact induced by the microfluidic environment.

Over the past decade, microfluidic chips, including the specific design used in our work, have become a widely accepted and powerful tool in microbial physiology research. A broad consensus has emerged within the community that the microenvironment within these microchannels does not significantly interfere with or perturb the natural physiological behavior of microorganisms (Dusny, C. & Grünberger, Curr Opin Biotechnol. 63, 26-33 (2020)). This understanding is also supported by the fact that key findings obtained with microfluidic single-cell technologies are reproducible by other methods. For example, the adder model of cell-size homeostasis in *E. coli* firstly observed in microfluidic chips has been repeatedly validated by different methods (Taheri-Araghi, S. et al. Curr. Biol. 25, 385-391 (2015)). Therefore, while we acknowledge the importance of considering environmental effects, we are confident that the synchronization we report reflects the genuine biological dynamics of *E. coli* cells.

(4) To further test and generalize the model, it would be informative to also examine bacterial responses at intermediate temperatures rather than focusing primarily on a single cold-shock condition.

We thank the reviewer for this thoughtful suggestion. In designing our experiments, we aimed to study the bacterial cold shock response at the single-cell level. A key feature of this response is that it is typically triggered only when the temperature drops below a certain threshold within a short time duration. We therefore chose to lower the temperature from 37 °C to 14 °C as rapidly as possible. This approach allowed us to leverage the unique capabilities of LUNA while also providing an opportunity to explore this biological process in greater detail.

We agree that investigating bacterial responses across intermediate temperatures would be highly informative for understanding how temperature changes affect cellular physiology. However, this direction addresses a distinct scientific question that lies beyond the scope of the current work. We fully acknowledge its value and do have the intention to explore it in future studies.

**Recommendations for the authors:**

**Reviewer #1 (Recommendations for the authors):**

Major points:

(1) To strengthen the generality of the conclusions regarding cold shock response, it would be helpful to include a similar single-cell analysis of growth and division (cell size and concentration) for the 37°C to 12°C temperature shift. In this case, the experimental acclimation lasts ~5 hours, whereas the model predicts ~2 hours (Fig. S16a). Examining whether the model still holds or whether additional factors (e.g., further reductions in cell size) contribute to the observed OD decrease would clarify this discrepancy.

We thank the reviewer for this valuable suggestion. Our model for explaining the population-level OD dynamics during acclimation does not depend on single-cell time-lapse microscopy data. Instead, the single-cell inputs used for parameterization were obtained from flow cytometry measurements, which quantify population-wide single-cell distributions. Therefore, the model is not intrinsically restricted to a specific imaging-based experimental setup or to a particular temperature shift.

Most of the quantitative analysis presented in the manuscript focuses on the 37°C to 14°C transition, where the model shows strong agreement with experimental OD measurements. We selected this condition because it provides high-quality, internally consistent datasets at both the single-cell and population levels. However, the modeling framework itself is mechanistic and parameter-based, rather than temperature-specific. In principle, it can be applied to other temperature shifts, provided that the corresponding single-cell growth and state-transition parameters are experimentally determined.

Regarding the temperature shift from 37°C to 12°C, the model demonstrates good agreement with the experimental observation that acclimation lasts approximately 5 hours. The minor deviations in several data points during the acclimation period can be attributed to systematic errors in the measurement of cell concentration and volume, as illustrated in the lower panel of Figure S16a. We are open to extend our analysis to additional temperature shifts in future work to further validate the model’s generality.

(2) Related to weakness #2, it would be helpful for the authors to clarify their definition of "synchronization" and to provide additional explanation or evidence supporting this claim. In particular, further discussion of the data in Fig. 4f, 4g, and S14 could help strengthen the proposed hypothesis.

We thank the reviewer for this constructive suggestion. In previous response (public review weakness #2), we clarified the definition of “synchronization” in the revised manuscript by explicitly distinguishing between two types of synchrony: (i) the synchrony in the timing of growth rate changes after cold shock, and (ii) the synchrony in division cycle progression. For the latter, we now use the more precise term “progression of division cycle” to avoid ambiguity. Furthermore, we have expanded the discussion of the data in Figures 4f, 4g, and S14 to better support the claim that cells actively maintain alignment in cycle progression. We hope these revisions address the reviewer’s concern and strengthen the evidence for our hypothesis.

Minor points:

(1) Line 78: "... and concluded that the OD lag is actually the outcome of the synergy of changes in bacterial concentration and volume, ..." The term synergy usually implies a combined effect greater than the sum of individual effects. Are the changes in bacterial concentration and volume synergistic here?

We agree with your observation that the term "synergy" in scientific contexts typically implies an interaction effect that is greater than the sum of individual effects. In our original phrasing, we intended to convey that the observed OD lag is a result of the combined contributions from both changes in bacterial concentration and changes in cell volume, rather than being dominated by a single factor. We did not mean to imply a super-additive interaction between these two variables.

We acknowledge that the relationship between bacterial concentration and cell volume can be complex and may even exhibit interdependence under certain conditions (e.g., under nutrient limitation at high OD). However, using "synergy" could indeed be misleading. To ensure terminological precision and avoid any potential misinterpretation, we will revise the text in the revised manuscript. We will replace "synergy" with a more neutral and accurate phrase "combined effect".

(2) Figure 2d: Why does the focusing time increase even after temperature stabilizes following the downshift? Does focus drift depend not only on rapid cooling but also on the lower steady-state temperature? Additional explanation would be helpful.

As noted in the Methods section ("Time-lapse imaging of bacteria under CS"), when the temperature was lowered, the objective lens heater was stopped, which caused a slightly longer focusing time. This is because prior to the temperature downshift, the objective heater maintained the objective at a temperature close to that of the sample (37°C), minimizing any thermal gradient between them. After the temperature decrease to 14°C, while the sample chamber was precisely controlled at the target low temperature, the objective lens now without active heating gradually equilibrated to ambient room temperature (approximately 22–25°C). This created a stable temperature mismatch between the relatively warmer objective and the colder sample. Such a temperature gradient can cause minor thermal expansion or contraction of the objective lens barrel, leading to a small but persistent shift in the focal plane. Consequently, the focusing time remained slightly elevated (∼0.6 s) compared to the 37°C condition (∼0.3 s), even after the sample temperature had stabilized. This offset reflects the steady-state thermal disequilibrium between the objective and the sample, rather than a transient cooling effect. We hope this explanation clarifies the reviewer’s concern.

(3) Line 234: "Reanalysis of the protein synthesis dynamics after CS revealed increase in CSPs synthesis (Figure 3e)." A citation is needed here. Additionally, the dataset referenced here was generated using a 37°C to 10°C cold shock.

We thank the reviewer for the insightful comments and the careful reading of our manuscript. We have now added the appropriate citation in the main text (Zhang, Y. et al. Molecular Cell 70, 274–286 (2018)). The dataset used in this reanalysis was generated under a 37°C to 10°C cold shock, rather than 12°C, and we have clarified this in the Methods section to avoid any ambiguity.

We would also like to clarify our rationale for using this published dataset in the present context. To our knowledge, no published dataset exists with comparable protein synthesis dynamics specifically at 12°C. Our intention here was to reference a well-characterized cold-shock dataset to support the qualitative point that CSP synthesis increases and ribosome synthesis decreases after cold shock. In cold shock studies, many qualitative conclusions are broadly consistent across low-temperature conditions (e.g., below ~15°C, and in some cases more broadly below ~20°C), including the observation that the ribosomal protein fraction is relatively insensitive to temperature change (Herendeen, S. L. et al. Journal of Bacteriology. 139, 185–194 (1979), Knapp, B. D. & Huang, K. C. Annual Review of Biophysics. 51, 499–526 (2022)). We appreciate the reviewer’s valuable feedback, which has helped us improve the clarity and accuracy of our work.

(4) Figure 3f and 3g: How is growth rate defined here, and why do the elongation rate and growth rate yield different results? My understanding is that, during steady-state growth, cell elongation rate increases as cells progress through a single cell cycle prior to division, whereas G0 cells exhibit reduced elongation rate following cold shock. Is this correct? More explanation is also needed for "linear growth in growth mode" (Line 267).

Thank you for this important comment. In our manuscript, we use:

Elongation rate = *dL/dt* (the absolute rate of increase in cell length; y-axis in Figure 3f)

Growth rate = (*dL/dt*)/*L* (i.e., *λ*, y-axis in Figure 3g; also referred to in some studies as the instantaneous growth rate)

Because these are different quantities, they do not necessarily follow the same trend across the cell cycle. To clarify the logic behind our “growth mode” classification (also see Willis & Huang, Nat Rev Microbiol 2017):

For a rod-shaped cell growing in length *L*,

(1) Exponential growth means the elongation rate is proportional to cell size, i.e.,

𝑑𝐿/𝑑𝑡 ∝ 𝐿

or equivalently,

((𝑑𝐿/𝑑𝑡)/𝐿) = constant

(2) Linear growth means the elongation rate is constant throughout the cell cycle, i.e.,

𝑑𝐿/𝑑𝑡 = constant

which implies that

((𝑑𝐿/𝑑𝑡)/𝐿)

decreases as the cell elongates.

Based on these two basic cases, additional growth modes (e.g., super-exponential, sub-exponential, sub-linear) can also be defined, as illustrated in the Author response image 1.

**Author response image 1.** 

With this definition, our interpretation of Figure 3f and 3g is as follows: before cold shock, cells are consistent with approximately exponential growth (red line in Figure 3g), whereas after cold shock, the G0 cells are better described as undergoing approximately linear growth (yellow line in Figure 3f).

(5) Figure S12: Why are the curves not continuous across GN, G0, G1, and G2?

In this figure, we present two different metrics: elongation rate (𝑑𝐿/𝑑𝑡) in panel (a) and growth rate (𝜆 = (𝑑𝐿/𝑑𝑡)/𝐿) in panel (b). During bacterial division, the cell length approximately halves while the growth rate remains constant under steady-state conditions. As a result, elongation rate, which is proportional to the instantaneous length, also halves at each division event, leading to the observed discontinuities at the time points corresponding to divisions (GN, G0, G1, and G2). In contrast, growth rate is inherently continuous across divisions, as shown in panel (b), although minor apparent discontinuities may appear due to the finite temporal resolution of our measurements. We hope this explanation clarifies the figure.

(6) Figure 4d: X-axis labels are missing.

Thank you for your insightful comment. The six panels share identical axes in Figure 4d. To enhance the visual focus on the data trends across different generations, we intentionally displayed the X-axis label and numerical tick labels only on the first panel. The subsequent panels show only the tick marks without the numerical labels, as their scale is identical to that of the first panel.

(7) Line 285 and Figure 4e: "The changes in *λ* are highly synchronized in time, with the exact time lag between any pair of ξ not exceeding 2 min ..." What is the definition of time lag?

In our study, the term "time lag" refers to the absolute difference in time at which a large sudden drop of the *λ* curve occurs between any two pairs of *ξ*. Essentially, it quantifies how closely the dynamic changes in *λ* are aligned across different groups. A time lag of zero would indicate perfect synchrony, while a value within 2 minutes implies that the variations in *λ* for any pair of *ξ* occur nearly simultaneously.

(8) Figure S14: Why can the elapsed cycles take negative values?

In Figure S14, we plotted the centered values. Specifically, at each time point, we calculated the mean elapsed cycle number across all lineages, and then subtracted this mean from each group’s value. The resulting values are presented in the figure as “Elapsed cycles (zero-centered)”. Thus, negative values are expected and meaningful they represent lineages that are progressing more slowly than the average at that time point. This transformation helps to highlight the relative differences among groups over time, while removing the overall temporal trend (which is already shown in Figure 4g).

(9) Figure 5 legend: Fitting for the acclimation has a R2 of -0.263 (Pearson correlation coefficient -0.00). R^2 should not be negative, and it doesn't agree with the calculated Pearson correlation coefficient.

Thank you for this important observation. Indeed, R^2^ should normally fall within the range [0, 1]. This discrepancy arises because the fitting model used differs from the default linear regression, and we did not specify this in the original figure legend. In the revised manuscript, this has been corrected. The explanation why R^2^ is negative here is as follows:

The linear fit used is *y* = *a*·*x* (i.e., no-intercept, forced through the origin). This is based on the physical principle that when OD is zero (no bacteria), the total bacterial mass must also be zero. For ordinary linear models with an intercept, R^2^ ranges from 0 to 1. However, for no-intercept models, the calculation of total sum of squares (*SS*_tot_) differs (typically relative to zero rather than the mean of y), and R^2^ can become negative if the fit performs worse than the baseline *y* = 0. Here, R^2^ = -0.263 simply indicates that for these specific data points, the origin-constrained linear fit does not outperform the trivial y=0 model. Regarding the Pearson correlation: The near-zero coefficient (-0.00) suggests no significant linear trend between *X* and *Y*, which is consistent with the poor fit performance.

(10) Language and typos: The manuscript contains grammatical errors and typos that require careful proofreading (one example: Line 56 "..., and reflection-based approaches ...").

We thank the reviewer for the careful reading and for drawing our attention to the language and typographical issues in the manuscript. In the revised version, we will carefully proofread the entire text and correct any errors and inconsistencies, including the example pointed out in line 56.

**Reviewer #2 (Recommendations for the authors):**

(1) The LUNA section is extremely technical and advanced for most biologists - it might be useful to include a few sentences in simple language why LUNA helps solve the biology question.

We thank the reviewer for the valuable suggestion. We have now added a concise, plain-language overview at the end of the LUNA section (Performance Analysis of LUNA):

“In brief, LUNA locks the focal plane with nanometer-scale precision over an ultra-large range rapidly, ensuring stable focus during long-term imaging for reliable observation of fine subcellular structures and dynamics.”

(2) The suggestions I included in the weakness section are not mandatory to perform, but will be helpful to at least discuss in the paper.

We thank the reviewer for the thoughtful comment and for acknowledging that the suggestions in the weakness section are not mandatory. We have carefully considered each point raised and have provided detailed responses in the point-by-point reply. While we recognize the potential value of these suggestions for further expanding the study, we respectfully believe that incorporating them into the current manuscript would go beyond the intended scope of this work.

Thanks

Otherwise, great job with the paper!

We are truly grateful to the reviewer for the encouraging feedback and appreciate the time and effort invested in improving our manuscript.
